# Utility of the PHQ-9 to identify major depressive disorder in adult patients in Spanish primary care centres

**DOI:** 10.1186/s12888-017-1450-8

**Published:** 2017-08-09

**Authors:** Roger Muñoz-Navarro, Antonio Cano-Vindel, Leonardo Adrián Medrano, Florian Schmitz, Paloma Ruiz-Rodríguez, Carmen Abellán-Maeso, Maria Antonia Font-Payeras, Ana María Hermosilla-Pasamar

**Affiliations:** 1 Department of Basic Psychology, Faculty of Psychology, University of Valencia, Av. Blasco Ibáñez, 21, 46010 Valencia, Spain; 2 Department of Basic Psychology, University Complutense of Madrid, Madrid, Spain; 3 Faculty of Psychology, University Siglo 21, Córdoba, Argentina; 4 Department of Psychology, Ulm University, Ulm, Germany; 5 Castilla La Nueva Primary Care Centre, Health Service of Madrid, Madrid, Spain; 6 Hospital Ntra. Sra. Perpetuo Socorro, Mental Health Service of Albacete, Albacete, Spain; 7 Hospital General de Villarrobledo, Mental Health Service of Albacete, Albacete, Spain; 8 Complejo Hospitalario Universitario of Albacete, Mental Health Service of Albacete, Albacete, Spain

**Keywords:** Major depressive disorder, Primary care, Patient health questionnaire-9, Psychometric properties

## Abstract

**Background:**

The prevalence of major depressive disorder (MDD) in Spanish primary care (PC) centres is high. However, MDD is frequently underdiagnosed and consequently only some patients receive the appropriate treatment. The present study aims to determine the utility of the Patient Health Questionnaire-9 (PHQ-9) to identify MDD in a subset of PC patients participating in the large PsicAP study.

**Methods:**

A total of 178 patients completed the full PHQ test, including the depression module (PHQ-9). Also, a Spanish version of the Structured Clinical Interview for DSM-IV Axis I Disorders (SCID-I) was implemented by clinical psychologists that were blinded to the PHQ-9 results. We evaluated the psychometric properties of the PHQ-9 as a screening tool as compared to the SCID-I as a reference standard.

**Results:**

The psychometric properties of the PHQ-9 for a cut-off value of 10 points were as follows: sensitivity, 0.95; specificity, 0.67. Using a cut-off of 12 points, the values were: sensitivity, 0.84; specificity, 0.78. Finally, using the diagnostic algorithm for depression (DSM-IV criteria), the sensitivity was 0.88 and the specificity 0.80.

**Conclusions:**

As a screening instrument, the PHQ-9 performed better with a cut-off value of 12 versus the standard cut-off of 10. However, the best psychometric properties were obtained with the DSM-IV diagnostic algorithm for depression. These findings indicate that the PHQ-9 is a highly satisfactory tool that can be used for screening MDD in the PC setting.

**Trial registration:**

Current Controlled Trials ISRCTN58437086. Registered 20 May 2013.

## Background

### Major depressive disorder in Spanish primary care

The vast majority of mental disorders in Spain are diagnosed in primary care (PC), which serves as a gateway to treatment and to the entire public health system [1]. In this context, emotional disorders are often misdiagnosed, with rates of up to 78% for depression, 71% for generalized anxiety disorder (GAD), and 86% for panic disorder [2]. Moreover, even among patients who are correctly diagnosed, only 35.8% of those with depression and 30.7% of those with any anxiety disorder receive adequate treatment [3] (i.e., most patients receive primarily pharmacological treatment, which is not recommended in clinical practice guidelines [4]). These mental disorders impose an important economic and societal burden on European countries, including Spain [5, 6].

Major depressive disorder (MDD) is highly prevalent in Spanish PC centres, with 9.6% of attendees suffering from this disorder each year [7], although this figure is lower than the mean prevalence rate (19%) in European countries [8]. Nevertheless, due to the absence of systematic screening tests, general practitioners (GPs) only recognize about 60% of cases of MDD [3], partly because this condition is frequently comorbid with other physical, somatic, and/or psychological problems such as anxiety disorders or alcohol abuse [9]. Based on international guideline recommendations (such as the NICE) to manage depression, it is clear that improved assessment methods (for both screening and diagnosis) are needed to improve MDD identification in order to refer these individuals to the appropriate therapeutic intervention [10]. For this reason, screening tests are very helpful to obtain a quick, initial identification of a possible case of MDD; however, such tools are not sufficiently reliable to be used as the sole detection instrument [10, 11]. Thus, clinical interviews are required as a second step to confirm diagnoses. The use of these screening tools followed by clinical interviews should increase the efficiency of PC centres and improve overall public health outcomes for MDD.

One screening test that could be used in PC centres to identify MDD is the PHQ-9 [12]. This self-report instrument is derived from the Primary Care Evaluation of Mental Disorders (PRIME-MD), which was originally developed to identify five mental disorders: depression, anxiety, alcohol abuse, somatoform disorder, and eating disorder. A systematic review of 16 studies that were carried out to identify depression [13] concluded that although there are many valid tools, the PHQ-9 is equal or superior to other instruments. In this context, given that the operating characteristics of these instruments are similar, selection of the optimal tool to identify MDD should depend on its feasibility, administration and scoring times, and the capability of the instrument to serve additional purposes, such as monitoring depression severity or response to therapy. Indeed, several meta-analyses recommended the PHQ-9 to identify depression in the PC setting because, it can be administered easily, quickly, and in a wide range of clinical contexts [14, 15]. For instance, Gilbody et al. [14] analysed 17 validation studies (> 5000 participants), concluding that the PHQ-9 has good psychometric properties (sensitivity 0.80, specificity 0.92) using either the ≥10 cut-off score or the “diagnostic algorithm” method. Manea, Gilbody and Mcmillan [15] analysed a total of 18 studies (7180 patients, 927 with MDD confirmed by diagnostic interviews), concluding that the PHQ-9 shows acceptable psychometric properties for MDD. In that study, using the widely-recommended cut-off score of 10, sensitivity was 0.85 and specificity 0.89, with no substantial differences in pooled sensitivity and specificity for cut-off scores ranging from 8 to 11.

The PHQ-9 items closely follow the nine criteria specified in the DSM-IV diagnostic manual (the core criteria for MDD have not changed in the DSM-5). Patients use Likert scales to rate the presence of symptoms in the prior two weeks. Depending on frequency (“not at all”, “several days”, “more than half of the days”, and “almost every day”), the nine items are scored from 0 to 3 points (total severity scores range from 0 to 27 points). Total scores of 10–14 points, 15–19 points, and 20–27 points indicate, respectively, moderate, moderately severe, and severe levels of depressive symptoms. When the PHQ-9 is used as a screening test, the most widely recommended cut-off value is 10, as previous research has demonstrated that this cut-off value provides the best combination of sensitivity (0.88) and specificity (0.88) [12]. The PHQ-9 has also been proposed for use as a diagnostic tool using a specific coding algorithm based on the DSM-IV criteria for MDD in which MDD is diagnosed if at least one of the two first symptoms (items) is rated with a 2 (more than half of the days) or a 3 (most days) and four of the remaining items are also rated with a score of 2 or 3 (with the exception of item 9 [suicide], in which a rating of 1 is sufficient). However, the general consensus is that the PHQ-9 can be used as a screening test but not as a diagnostic test [12–15].

The construct validity of the PHQ-9 has been demonstrated in PC patients in many countries, including Spain [16], Brazil [17], China [18], East-Africa [19], Holland [20], South-Africa [21], the US [22] and others. These studies indicate that the PHQ has a high convergent validity with other depression measures. However, questions have been raised with regard to the optimal cut-off scores for screening to obtain the most accurate results on the PHQ-9. For example, a meta-analysis [12] suggested that the PHQ-9 presented good screening properties with both the ≥10 cut-off and the “diagnostic algorithm” method, but that the cut-off point may be increased to ≥11 or ≥12 to obtain optimum specificity in some community-based studies. In a recent review, Kroenke et al. [23] argued against using an inflexible adherence to a single cut-off score; rather, those authors argue that the cut-off should be adjusted to the target population. Manea et al. [15] found no significant differences in sensitivity or specificity between a cut-off score of 10 and other cut-off scores (ranging from 8 to 11), but suggested that a cut-off of 11 may represent the best trade-off between sensitivity and specificity. Although the optimal cut-off point is controversial and may depend on the target population, the PHQ-9 presents a reasonably good sensitivity and specificity when used as a screening tool, regardless of the precise cut-off point. By contrast, in studies conducted to assess the validity of the “diagnostic algorithm”, results have been more ambiguous. A recent meta-analyses performed to assess 27 validation studies of the PHQ-9 algorithm scoring method in various settings concluded that—in most cases—sensitivity was low but specificity was good [24]. Similarly, Mitchell et al. [25] conducted a meta-analysis of 26 publications reporting on 40 individual studies (*n* = 26,902 patients), finding that the best estimates of sensitivity and specificity for the PHQ-9 algorithm were 0.57 and 0.93, respectively. So, the PHQ-9 can be used as a screening test using different cut-off scores but the psychometric properties of the “diagnostic algorithm” were not as good.

Few studies have evaluated the Spanish version of the PHQ-9. The first study by Diez-Quevedo et al. [26] was conducted to validate the Spanish version of the whole PHQ (including the 9 items for depression) in an inpatient setting, finding that this 9-item part of the PHQ-9 yielded satisfactory sensitivity (0.84) and excellent specificity (0.92) for MDD compared to the gold standard at that time (i.e., the Structured Clinical Interview for DSM-III-R). However, the profile of patients in PC centres is likely to differ substantially from those treated in a psychiatric inpatient setting. A Spanish version of the PHQ-9 has also been evaluated for use in PC centres in Honduras, with all of the linguistic and cultural differences implied by that setting [27]. However, only one study has focused on a Spanish version of the PHQ-9 for Spain [16]. In that study, although the sample was obtained from Spanish PC centres, the PHQ-9 was administered by telephone, and thus reported internal consistency of the PHQ-9 applies only to telephone administration. Consequently, little is known about how the PHQ9 performs in Spanish PC centres, nor do we know the optimal cut-off criteria that would be most appropriate in this context in Spain.

### Objectives

The aim of the present study was to assess the utility of the PHQ-9 as a screening test to identify MDD in patients at Spanish PC centres. We performed psychometric analyses to identify the sensitivity and specificity of the PHQ-9 total score to obtain the optimal cut-off value based on diagnoses obtained with the standardized clinical interview (Structured Clinical Interview for DSM-IV Axis I Disorders; SCID-I). Additionally, we tested sensitivity and specificity of the “diagnostic algorithm”.

## Methods

### Setting

The study was conducted from January to December 2014 at five PC centres participating in the larger PsicAP study [28], a clinical trial designed to evaluate the diagnosis and treatment of emotional disorders among PC patients in Spain. The centres are located in several cities in Spain (two in Valencia, and one each in Albacete, Vizcaya, and Mallorca).

### Instruments

#### Patient health questionnaire (PHQ)

The PHQ is a self-report screening test derived from the PRIME-MD test [29]. The PHQ also includes modules to assess somatization (PHQ-15), depressive disorder (PHQ-9), panic disorder (PHQ-PD), generalized anxiety disorder (GAD-7), eating disorders, and alcohol-related disorders. In this study, we used the Spanish GAD-7 validation by García-Campayo et al. [30], which contains the 7 GAD items.

#### PHQ-9

The PHQ-9 [12] is part of the PHQ and consists of nine items to assess for the presence of the nine diagnostic criteria for major depression according to DSM-IV. The PHQ-9 evaluates the presence of the following symptoms over the previous two-week period: (a) depressed mood; (b) anhedonia; (c) sleep problems; (d) feelings of tiredness; (e) changes in appetite or weight; (f) feelings of guilt or worthlessness; (g) difficulty concentrating; (h) feelings of sluggishness or worry; (i) suicidal ideation. Items are answered on a four-point Likert scale from 0 to 3 as follows: 0 (never), 1 (several days), 2 (more than half of the days), and 3 (most days). Internal consistency was satisfactory in the current sample (McDonald’s *ω* = .89) and all item-test correlations were >.40. A public version of the PHQ-9, written in Spanish for use in Spain, provided by the authors of the PHQ was used in this study.

#### Structured Clinical Interview for DSM-IV Axis I Disorders (SCID-I)

The Spanish Version of this semi-structured interview [31] was conducted by clinical psychologists (7 in total) who had received intensive training by an expert clinical psychologist (see Cano-Vindel et al. [28] for more details). The interview sessions were supervised by the same clinical psychologist for the duration of the study. Patients were diagnosed with MDD when they presented at least five of the DSM-IV criteria during the last two weeks: that is ≥ one of the first two symptoms and ≥ four of the remaining symptoms.

### Procedure

Patients with anxious, depressive or physical symptoms without a clear biological basis were asked by the GPs to participate in the PsicAP clinical trial (see Cano-Vindel et al. [28]). They received the Patient Information Sheet and provided informed consent. Next, an individual meeting was arranged to review the study details with the participants and to complete the PHQ and the other tests. Computerized versions of these tests were used in most cases. Patients with impaired vision received help in completing the questionnaires. Paper versions of the measures were provided to patients with difficulties using the computer. After completing the PHQ-9, participants were asked to participate in the SCID-I interview, which was then scheduled within a maximum of 2 weeks from completion of the PHQ-9. Prior to administration of the SCID-I, all participants received a Patient Information Sheet of this sub-study and signed an informed consent form. All clinical psychologists conducting the interviews were blinded to the results of the PHQ-9.

This study was approved by the Corporate Clinical Research Ethics Committee of Primary Care of Valencia (CEIC-APCV) (as the national research ethics committee coordinator) and the Spanish Medicines and Health Products Agency (AEMPS) (N EUDRACT: 2013–001955-11 and Protocol Code: ISRCTN58437086).

### Data analysis

A receiver operating characteristic (ROC) curve analysis was performed using data from the 178 patients that completed the PHQ-9 and were interviewed with the SCID-I; this statistical analysis was performed using the pROC package [32] for the statistical programming environment R [33]. The following ratios were calculated: sensitivity, specificity, positive and negative predictive values, and positive and negative likelihood ratios. To evaluate the test’s screening properties, we used the sum scores of the PHQ-9 and the “diagnostic algorithm”. The optimal cut-off value to balance sensitivity and specificity was defined as the value corresponding to the maximum value of the Youden’s index, calculated as (sensitivity + specificity – 1) [34].

## Results

### Study sample

All patients between 18 and 65 years (inclusive) who presented at one of these five PC centres for somatic or psychological complaints during the study inclusion period were invited to participate (*n* = 298). Of these, 260 participants (186 females) completed the PHQ and 178 (125 females) were interviewed using the SCID-I. In terms of socio-demographic variables, no differences were observed between the whole sample and the subset of participants who completed the SCID-I interview (as indicated by t-tests or chi-squared tests, depending on variable type; all *p* ≥ .35). The Vizcaya centre, however, had a slightly higher dropout rate. Table 1 shows the socio-demographic variables and data on prescription medications taken by the patients.

**Table 1:** Demographics and medication

	Total sample of PHQ respondents (*n* = 260)		Subsample of PHQ and SCID-I respondents (*n* = 178)	
	*n*	%	*n*	%
Primary Care Centre				
Albacete	39	15.0	21	11.8
Mallorca	33	12.7	30	16.9
Valencia	155	59.6	122	68.5
Vizcaya	33	12.7	5	2.8
Sex				
Female	186	71.5	125	70.2
Male	74	28.5	53	29.8
Marital status				
Married	130	50.0	86	48.3
Divorced	28	10.8	21	11.8
Widowed	5	1.9	3	1.7
Separated	19	7.3	14	7.9
Never married	48	18.5	29	16.3
Unmarried	30	11.5	25	14.0
Level of education				
No schooling	7	2.7	4	2.2
Basic education	94	36.2	71	39.9
Secondary education	40	15.4	27	15.2
High School	64	24.6	46	25.8
Bachelor	47	18.1	27	15.2
Master/doctorate	8	3.1	3	1.7
Employment situation				
Part-time employee	28	10.8	18	10.1
Employed full time	85	32.7	58	32.6
Unemployed, in search of work	77	29.6	52	29.2
Unemployed, not looking for work	36	13.8	27	15.2
Temporary low labor	14	5.4	11	6.2
Permanent low labor	4	1.5	2	1.1
Retired	16	6.2	10	5.6
Income level				
Less than 12,000	119	45.8	87	48.9
12,000 to 24,000	112	43.1	79	44.4
Between 24,000 and 36,000	20	7.7	10	5.6
More than 36,000	9	3.5	2	1.1
Hypnotics				
No	147	56.5	100	56.2
Yes	113	43.5	78	43.8
Anxiolytics/tranquilizers				
No	175	67.3	119	66.9
Yes	85	32.7	59	33.1
Anti-depressants				
No	194	74.6	126	70.8
Yes	66	25.4	52	29.2

### SCID-I-based prevalence

Of the 260 patients included in our study, 178 completed the clinical interviews with the SCID-I. The prevalence of MDD seen in our PC population was high: 129 of 178 patients (72.5%) met the criteria for MDD on the SCID-I, while 49 patients (27.5%) did not fulfil these criteria.

### PHQ-based prevalence

Of the 260 patients who completed the PHQ, 141 (54%) met the criteria for somatization disorder (SD; (PHQ-15 ≥ 5), 68% for MDD (*n* = 178) according to the DSM-IV “diagnostic algorithm” or 78% PHQ-9 for scores ≥10 (*n* = 203) and 69% for GAD (GAD-7 ≥ 10; *n* = 180). 110 participants (42%) met the criteria for panic disorder according to the modified algorithm of the PHQ-PD and 22% (*n* = 57) met panic disorder criteria according to the original algorithm of the PHQ-PD. Finally, 17% (*n* = 45) met criteria for eating disorder) and 14% (*n* = 38) for alcohol-related disorder. As expected, comorbidity between disorders was high, particularly for comorbid MDD and GAD (*n* = 150; 57%), SD and MDD (*n* = 115; 44%), and GAD and SD (*n* = 117; 45%). Overall, 40% of the participants with MDD presented comorbidity with either GAD or SD (*n* = 104). We found no differences between the total sample of PHQ-9 respondents (*n* = 260) and the subsample of PHQ-9 and SCID-I respondents (*n* = 178) in terms of the proportion of participants that met criteria for one or more of the aforementioned disorders, nor with regard to comorbidities (all *p* > .61). See Table 2 for details.

**Table 2:** PHQ-based prevalence and comorbidity

	Total sample of PHQ respondents (*n* = 260)		Subsample of PHQ and SCID-I respondents (*n* = 178)	
	*n*	%	*n*	%
Somatoform disorder (SD)				
SD (≤ 5)	141	54.2	94	52.8
Major depressive disorder (MDD)				
MDD (Algorithm)	178	68.5	124	69.7
MDD (≤ 10)	203	78.1	138	77.5
Panic disorder (PD)				
PD (Original Algorithm)^a^	57	21.9	40	22.5
PD (Modified Algorithm)^b^	110	42.3	74	41.6
General anxiety disorder (GAD)				
GAD (≤ 10)	180	69.2	128	71.9
Eating disorder				
(PHQ Algorithm)	45	17.3	30	16.9
Alcohol abuse				
(PHQ Algorithm)	38	14.6	25	14.0
Comorbidity				
MDD + GAD	150	57.7	107	60.1
MDD + SD	115	44.2	81	45.5
GAD + SD	117	45.0	81	45.5
MDD + GAD + SD	104	40.0	74	41.6
GAD + PD	45	17.3	33	18.5
MDD + PD	40	15.4	30	16.9
MDD + GAD + PD	37	14.2	29	16.3
PD + SD	42	16.2	27	15.2
SD + GAD + PD	36	13.8	25	14.0
MDD + SD + PD	34	13.1	23	12.9
SD + MDD + PD + GAD	32	12.3	22	12.4
SD + MDD + PD + GAD	1	0.4	1	0.3
+ Eating + Alcohol				

*Note: SD* somatoform disorder, *MDD* major depressive disorder, *PD* panic disorder, *GAD* general anxiety disorder, *Eating* eating disorder, *Alcohol* alcohol abuse. Comorbidity categories are not exclusive (e.g., “MDD + GAD” comprises “MDD + GAD + SD”)

^a^Original Algorithm: All of the first four questions are answered with “yes,” and presence of four or more somatic symptoms during an anxiety attack

^b^Modified Algorithm: At least two of the first four questions are answered with “yes,” other coding criteria unchanged. (See Muñoz-Navarro et al. for more details; [35])

### Operating characteristics of the PHQ-9 using different cut-off scores

**Fig. 1:**
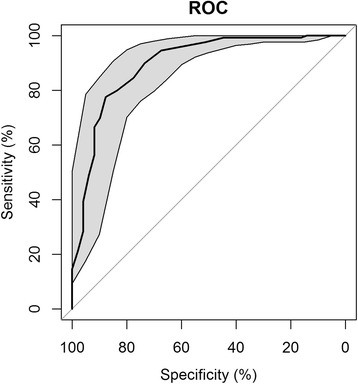
ROC curves for the PHQ-9 scale

The ROC curve analysis showed that the PHQ-9 had an area under the curve of 0.89 (Fig. 1). The most widely used cut-off value for correctly identifying cases with MMD is ≥10. In our study, of the patients diagnosed with MDD according to SCID-I, 95% had scores >10 on the PHQ-9 while 67% of patients without a SCID-I diagnosis of MDD scored below the cut-off level (< 10). As a result, the PHQ-9 had a sensitivity of 0.95, a specificity of 0.67, positive and negative predictive values of 0.88 and 0.83, respectively, and positive and negative likelihood ratios, respectively, of 2.90 and 0.08. Increasing the PHQ-9 cut-off point to 12 yielded the following values: sensitivity, 0.84; specificity, 0.78; positive and negative predictive values of 0.91 and 0.66, respectively; and positive and negative likelihood ratios of 3.76 and 0.20, respectively. Most (84%) depressed patients (SCID-I diagnosis) had scores of 12 or higher, whereas 78% of patients without a depression diagnosis scored below the cut-off point. Moreover, according to the Youden’s index, which offers the optimal cut-off value balancing sensitivity and specificity (sensitivity + specificity – 1), the most appropriate cut-off value was 14 (J = 0.66), whereas these values were lower when other cut-off scores were used, as follows: 10 (J = 0.62), 11 (J = 0.63), 12 (J = 0.62). With a cut-off score of 14, the PHQ-9 showed the following psychometric properties: sensitivity, 0.78; specificity, 0.88; positive and negative predictive values, 0.94 and 0.60, respectively; and positive and negative likelihood ratios, 6.33 and 0.26, respectively (Table 3 shows other possible cut-off points and confidence intervals).

**Table 3:** PHQ-9 operational characteristics

Cut-off Score	Sensitivity	Specificity	Positive Predictive Value	Negative Predictive Value	Positive Likelihood Ratio	Negative Likelihood Ratio	Youden’s Index (J)
PHQ-9 ≥ 8	.98 (.94–.99)	.51 (.37–.64)	.84	.89	1.99 (1.50–2.66)	.05 (.01–.14)	.49
PHQ-9 ≥ 9	.96 (.91–.98)	.59 (.45–.72)	.86	.85	2.36 (1.68–3.30)	.07 (.03–.16)	.55
PHQ-9 ≥ 10	.95 (.89–.97)	.67 (.53–.79)	.88	.83	2.90 (1.93–4.34)	.08 (.04–.17)	.62
PHQ-9 ≥ 11	.90 (.84–.94)	.73 (.60–.84)	.90	.73	3.39 (2.11–5.42)	.14 (.08–.24)	.63
PHQ-9 ≥ 12	.84 (.77–.90)	.78 (.64–.87)	.91	.66	3.76 (2.22–6.37)	.20 (.13–.31)	.62
PHQ-9 ≥ 13	.80 (.72–.86)	.84 (.71–.91)	.93	.61	4.89 (2.58–9.27)	.24 (.17–.35)	.64
PHQ-9 ≥ 14	.78 (.70–.84)	.88 (.76–.94)	.94	.60	6.33 (2.98–13.47)	.26 (.18–.36)	.66
Algorithm^a^	.88 (.82–.93)	.80 (.66–.88)	.92	.72	4.33 (2.48–7.55)	.15 (.09–.24)	.68

^a^MDD is diagnosed if at least one of the first symptoms (items) is rated with a 2 (more than half of the days) or a 3 (most days)

### Operating characteristics of the PHQ-9 using the “diagnostic algorithm”

Of the patients with a SCID-I diagnosis of MDD, 88% were also identified as having major depression according to the PHQ-9 “diagnostic algorithm”. By contrast, 80% of non-depressed patients (SCID-I) did not reach the diagnostic cut-off point. Based on these data, the PHQ-9 presented a sensitivity of 0.88, a specificity of 0.80, positive and negative predictive values, respectively, of 0.92 and 0.72, and positive and negative likelihood ratios of 4.33 and 0.15, respectively. The highest value for the Youden’s index (J = 0.68) was obtained for the PHQ-9 “diagnostic algorithm”. (Table 3 provides mores details, including confidence intervals and alternative cut-off points).

## Discussion

In this study, we assessed the utility of the PHQ-9 as a screening tool to identify MDD in users of Spanish PC services. The main appeal of the PHQ-9 is that it is an easy to administer and inexpensive self-report measure. Our main finding is that the PHQ-9 is of value in identifying MDD in patients at Spanish PC centres, but our findings suggest that a higher cut-off value (12 or more) or the “diagnostic algorithm” might be better than the standard 10-point cut-off value in order to improve specificity in this patient population.

Our results show that the PHQ-9 is a sensitive screening instrument for MDD, and in most cases it correctly identified individuals with MDD when the most common cut-off point (10 points) was used [12, 13, 35]. Unexpectedly, the specificity of the PHQ-9 in our study was much lower than reported in previous studies, suggesting more false positive diagnoses of MDD. Increasing the cut-off point to 12 resulted in a slight decrease in sensitivity but specificity improved to a more satisfactory value, yielding a more acceptable trade-off. At the 12-point cut-off, the positive predictive value increased while the negative predictive value decreased. According to the Youden’s index, the most appropriate cut-off score was 14 (J = 0.66) compared to a cut-off score of 10 (J = 0.62), 11 (J = 0.63), 12 (J = 0.62). Using a cut-off point of 14, the sensitivity was 0.78 and the specificity 0.88. To reduce false negatives, an important characteristic of a good screening tool is a high sensitivity. For this reason, we suggest a cut-off score of 12 in the context of Spanish PC centres due to the high sensitivity (0.84) achieved with this cut-off level. However, the optimal cut-off in other populations may vary and other authors have recommended adjusting the cut-off point to suit the target population [13, 14]. Given that sensitivity is vital in the PC setting, we believe that a moderate specificity (found in the cut-off score of 10) is acceptable. Thus, rather than strictly following the Youden’s index, we believe that our recommendations are more appropriate for clinicians in this setting.

Using the original DSM-IV algorithm to identify MDD, the results of the PHQ-9 were satisfactory, with a very high sensitivity (0.88) and good specificity (0.80). Consequently, the positive predictive value was quite high, the negative predictive value was acceptable, and the positive and negative likelihood ratios were, therefore, also good. Moreover, the Youden’s index showed the best index value (J = 0.68) when using the “diagnostic algorithm” compared to other cut-off scores. Overall, these results indicate that, from a psychometric perspective, the DSM-IV “diagnostic algorithm” is superior to most common cut-off scores of 10 or the other suggested values (ranging from 12 to 14 points), with an excellent ability to correctly differentiate between depressed and non-depressed individuals. Furthermore, the satisfactory positive and negative predictive values of the PHQ-9 show that the test is excellent for ruling out non-MDD cases but can also adequately confirm MDD. These findings are also consistent with the Spanish validation study [26], which also found high sensitivity and specificity under these conditions, as the “diagnostic algorithm” was used in the depression section. Based on these findings, we believe the DSM-IV algorithm should be used with the PHQ-9. In contrast to some previous research [24, 25, 36], these results suggest that the PHQ-9 can be used as a screening test when the DSM-IV “diagnostic algorithm” is used. That said, it is important to stress that the “diagnostic algorithm” used for screening purposes should not be confused with a diagnosis of MDD. We agree with Mitchell et al. [25] that the PHQ-9 should not be used as the only source of information to confirm a clinical diagnosis. Thus, the “diagnostic algorithm” for the PHQ-9 may serve as a useful screening method to quickly and efficiently identify MDD or other depressive symptoms in the PC setting. However, patients with suspected MDD should be referred for a clinical interview performed by an experienced clinician to confirm the diagnosis and to determine secondary causes.

This study presents some limitations that may have contributed to the discrepant results compared with other studies. To start with, patient recruitment required a referral by the GP, who informed patients about this clinical trial involving psychological treatment. This recruitment approach likely resulted in some degree of selection bias, which may have partially affected our results. This influence may have been negative because it seems probable that the low specificity of the PHQ-9 observed in our sample using a 10-point cut-off value may be attributable to some participants exaggerating their symptoms on the questionnaire to ensure eligibility for treatment. This hypothesis is supported by the fact that many patients with scores >20 (indicative of severe depression) were diagnosed as only mildly depressed on the SCID-I interview. Additionally, in previous studies, patients scoring >20 on that test did not present severe MDD [37]. In fact, based on those findings, Zimmerman et al. [37] called for caution in using the PHQ-9 to guide treatment selection until the thresholds to define severity ranges have been empirically established. Importantly, based on these findings, we have since modified the protocol of the PsicAP study [28] to prevent misuse: patients with PHQ-9 scores above 20 are automatically interviewed with the SCID-I to confirm the severity of their depression. Another limitation is that many patients that participated in our study presented symptoms of other emotional disorders, such as anxiety, somatizations, and mood disorders. Given that anxiety and depression share common features [38], this may explain the high rates of comorbidity. Thus, it is possible that patients suffering from anxiety or somatizations may have depressive symptoms that did not meet DSM-IV criteria for MDD on the SCID-I. In turn, this would have affected specificity estimates in our data. In fact, it is possible that the “diagnostic algorithm” performed better than other cut-off values because it is better adapted to these circumstances that are typically observed in the applied clinical setting. Therefore, the PHQ-9 may have some ecological validity for PC settings, where comorbidity is high and resources and available time are scarce. However, more studies are needed in Spanish PC centres to replicate these results and to identify possible boundary conditions. Additionally, given that the DSM-5 and DSM-IV use the same algorithm to diagnose MDD, a fertile area for future research would be to investigate the relationship between the PHQ and the restructured broader diagnoses of DSM-5 affective disorders.

## Conclusions

This is the first study to assess the PHQ-9 to obtain the optimal cut-off values for screening patients with MDD in the PC setting in Spain. The findings presented in this study indicate that the PHQ-9 is a valuable tool to help to identify suspected cases of MDD among patients treated at Spanish PC centres. Based on our results, in this population we recommend using a cut-off value of 12 or the DSM-IV “diagnostic algorithm” instead of the most common cut-off value of 10. Patients identified by the PHQ-9 screening tool with suspected MDD must be referred to specialised clinicians to confirm the diagnosis with other diagnostic measures and/or clinical interviews.

## Abbreviations

AEMPS: Spanish Medicines and Health Products Agency
CEIC-APCV: Corporate Clinical Research Ethics Committee of Primary Care of Valencia
DSM-5: Fifth Edition of the Diagnostic and Statistical Manual of Mental Disorders
DSM-IV: Fourth Edition of the Diagnostic and Statistical Manual of Mental Disorders
GAD: Generalized anxiety disorder
GAD-7: 7-item Generalized Anxiety Disorder
GP: General practitioner
MDD: Major depressive disorder
PC: Primary care
PD: Panic disorder
PHQ: Patient Health Questionnaire
PHQ-15: 15-item Patient Health Questionnaire
PHQ-9: 9-item Patient Health Questionnaire
PHQ-PD: Patient Health Questionnaire-Panic Disorder
PRIME-MD: Primary Care Evaluation of Mental Disorders
PsicAP: Psicología en Atención Primaria
ROC: Receiver operating characteristic
SCID-I: Structured Clinical Interview for DSM Axis-I Disorders
SD: Somatoform disorder
